# Dietary practices among individuals with diabetes and hypertension are similar to those of healthy people: a population-based study

**DOI:** 10.1186/s12889-015-1801-7

**Published:** 2015-05-10

**Authors:** Silvia GI Ozcariz, Carla de O Bernardo, Francieli Cembranel, Marco A Peres, David A González-Chica

**Affiliations:** Post-Graduate Program in Public Health, Federal University of Santa Catarina, 88040-970 Florianópolis, SC Brazil; Australian Research Centre for Population Oral Health. School of Dentistry, The University of Adelaide, Adelaide, South Australia Australia; Post-Graduate Program in Nutrition, Federal University of Santa Catarina, Florianópolis, SC Brazil

## Abstract

**Background:**

Currently, diabetes mellitus (DM) and systemic arterial hypertension (SAH) are among the top five global risks for mortality. Among the modifiable factors, careful dietary practice is one of the essential elements for the control of NCDs, since these diseases are often the result of unhealthy lifestyles. Thus, this study aimed to assess the frequency of dietary practices among adult males and females with DM and/or SAH, and compare whether or not they are more frequent than in healthy adults, through a population-based study conducted in the city of Florianópolis, southern Brazil.

**Methods:**

Cross-sectional population-based study, using as exposure self-reported DM and/or SAH status. Dietary practices were assessed using a semiquantitative food consumption questionnaire. The following were considered as adequate: regular intake (≥ 6 times/week) of fruit and vegetables, daily intake of fruit (≥ 3 times/day) and vegetables (≥ 2 times/day), intake lower than 2 times/week of meat fat, fried foods, and soda. Bivariate and adjusted analysis for sociodemographic variables were conducted using Poisson regression, stratified by gender.

*Location*: Florianópolis, southern Brazil, 2009.

*Subjects*: Representative sample of 20 to 59 year-old adults (n=1720).

**Results:**

A total of 16.6% participants were diagnosed with DM and/or SAH. The most frequently consumed unhealthy foods were fried food (51.0%, 95% CI: 48.8–53.5) and soda (57.9% 95%CI: 55.5–60.2). Of healthy foods, fruit was the less consumed on a daily basis (11.1% 95%CI 9.6-12.5). In general, women showed better dietary practices than men. In adjusted analysis none of dietary practices was more frequent among diabetic and/or hypertensive adults compared with healthy individuals, regardless of gender. No differences were found between healthy and unhealthy adults, when the number of dietary practices was assessed.

**Conclusions:**

The frequency of dietary practices was low and did not differ between individuals with or without DM and/or SAH. It is fundamental to reinforce the need of healthy dietary practices as one of the essential elements for the control of chronic diseases and their complications.

## Background

Since the 1970s, Brazil has followed the global trend of demographic, nutritional, and epidemiological transition, which has caused major changes in the pattern of disease occurrence in the population. These processes have brought along an increased prevalence of overweight [1] and non-communicable diseases (NCDs), especially diabetes *mellitus* (DM) and systemic arterial hypertension (SAH) [1].

DM is a major growing public health problem [2]. In 2012, the World Health Organization (WHO) reported that, according to estimates and in a worldwide range, one in ten adults carries DM [3] (mostly type 2), and this number will double by 2025 [4]. In Brazil, it is estimated that 5.6% of the population over 18 years old has been diagnosed with this disease [5]. SAH is another major chronic illness, and it is one of the most frequent health problems nowadays [3]. SAH has increasingly reached more people, and it is estimated to affect one in three people over 18 years of age worldwide [3]. The prevalence of SAH among adults in Brazil ranges from 22.3% to 43.9% [6]. These values are similar to high-income countries, such as the United States, where in 2008 the prevalence was estimated in 34% in adults [7].

Currently, DM and SAH are among the top five global risks for mortality [3]. Because DM and SAH are chronic diseases with severe complications and are difficult to control, they end up being costly for the individual, their families and for health services [8]. In Brazil, these two NCDs are responsible for the largest share of hospital care costs in the National Health System [9-11].

The treatment of these diseases includes not only drug intervention, but mostly a change in lifestyle. Among the modifiable factors, careful dietary practice is one of the essential elements for the control of NCDs, since these diseases are often the result of unhealthy lifestyles. WHO data indicates that about 80% of DM and SAH cases could be avoided through the adoption of dietary practices, such as regular consumption of fruit and vegetables, reduced consumption of saturated fats, sodium and sugary drinks, as well as increased physical activity and control of smoking habits [12]. Faced with such evidence, secondary prevention is essential, since it can positively change the evolutionary history of these problems, improving prognosis, life expectancy and quality [2,13]. However, success in controlling these diseases depends on adequate knowledge and patient decision to change his lifestyle [14]. In the United States, the National Health and Nutrition Examination Survey (NHANES) [15] investigated nearly 6,000 people from 2003 to 2004 and 2005 to 2006, and found no difference in the dietary practices of people with or without DM and/or SAH. In Brazil, it was not found any population based study in the scientific literature assessing if dietary practices are more common among people diagnosed with these NCDs. This kind of information is essential for the health planning of middle-income countries, such as Brazil, considering the quick process of nutritional and epidemiological transition they go through.

Thus, this study aimed to assess the frequency of healthy and unhealthy dietary practices among adult males and females with DM and/or SAH, and compare whether or not they are more frequent than in healthy adults, through a population-based study conducted in the city of Florianópolis, southern Brazil.

## Methods

Cross-sectional population-based study conducted with a representative sample of adults living in the city of Florianópolis, capital of the state of Santa Catarina, southern Brazil. The estimated population in 2009 was 408,161 inhabitants [16], and the city had a human development index of 0.847 in 2010 (third best in Brazil) [17].

The results of this paper are part of the EpiFloripa Adulto survey, conducted from September 2009 to January 2010. This survey investigated several general and oral health conditions, anthropometry, habits and usage of health services in a representative sample of the 249,530 20–59 year-old adults living in the urban area of Florianópolis [16].

The sampling process was performed in two phases. Initially, 60 of the 420 census sectors of Florianopolis were drawn. The number of households in each one of the census sectors was updated. Then 18 households were selected in each of the sectors, aiming to reach the expected size of the sample (n = 2016). Data collection was undertaken by trained interviewers from September 2009 to January 2010. Personal Digital Assistants were used to apply face-to face interviews. More details about sampling process and inclusion/exclusion criteria can be assessed elsewhere [18].

Quality of the information was evaluated by phone calls to approximately 15% of the sample (n = 248), applying a reduced questionnaire consisting of 10 questions. Kappa values and intra-class correlation ranged between 0.6 and 0.9 (0.7 for a lifestyle question).

### Independent variable

Considering the objectives of this study the independent variable was defined by the following questions: “Has a doctor or health professional ever said that you have diabetes?”; and “Has a doctor or health professional ever said that you have hypertension (high blood pressure)?”. The answers to both questions were grouped, and the exposure variable was established: diagnosed with DM and/or SAH (yes/no).

### Dependent variables

Variables related to healthy and unhealthy dietary practices were collected based on a semiquantitative food questionnaire including 18 questions on weekly and daily frequency of food consumption, in accordance with a model used in a Brazilian nationwide study entitled Surveillance of Risk and Protective Factors for Chronic Diseases on Telephone Interviews [19]. Monteiro et al. research [20] shows that indicators of food consumption used by this system are reproducible (Kappa between 0.6 and 0.8) and compared to three 24-hour recalls, have adequate validity for most indicators (sensitivity and specificity of ~ 80% for unhealthy food consumption and 42–80% for healthy food consumption). Based on these questions the following dichotomous variables of healthy food consumption (yes/no) were determined: regular consumption of fruit, regular consumption of vegetables, daily intake of ≥ 3 times/day of fruit and; daily consumption of ≥ 2 times/day of vegetables [21]. Positive diagnosis of these four variables referred to six or more days/week, and the two regular consumption variables were created regardless of how often these foods were consumed daily. In turn, to assess unhealthy food consumption the following dichotomous variables (yes/no) were used: regular consumption of meat fat (fat of red meat or chicken), regular consumption of fried foods, and; regular consumption of soda with added sugar (all considered as unhealthy foods). Regular unhealthy food consumption referred to the consumption of such foods two or more times/week (daily frequency intake data was not collected). Additionally, it was created a healthy dietary practice score based on the food guide for the Brazilian population [21], considering the above-mentioned matters. A point was scored for the daily consumption of each one of the healthy foods (≥3 times/day fruit and ≥ 2 times/day vegetables) and one point for those who did not eat on ≥ 2 days/week each of the unhealthy foods (meats fat, fried foods and soft drinks). Thus a scale from zero (no healthy eating behaviour) to five points (complete healthy eating behaviour) was established. For result exhibition and graphs, score points were grouped as follows: 0–1 point, 2–3 points, 4–5 points.

There were also considered as possible confounders: age in full years (20–29 years, 30–39 years, 40–49 years and 50–59 years-old), self-reported skin colour (white, dark, black, and other), education level in completed years of study (0–8 years, 9–11 years and ≥ 12 years) and per capita income in Reais (R$) (upper tertile = 1300.10 to 33333.00; intermediate tertile = R$ 567.00 to 1300.00; lower tertile = up to R$ 566.90 – 1 USD = 1.72 R$ in 2009). Considering the evidences of different food habits in men and women [22,23], as well as the different access to health services [24,25], all the analyses were stratified by gender, assuming that this variable is a potential effect modifier in the associations between the disease status and the dietary practices.

Data analysis was conducted using the 11.0 Stata program, always considering the process of cluster sampling and respective sample weights (Stata “svy” command). Descriptive analyses were performed by calculating relative frequencies and their 95% confidence intervals (95% CI). Chi-square with Rao-Scott correction was used for bivariate analyses. Venns diagram was used to show the frequency of dietary practices in relation to consumption of healthy and unhealthy food between gender and disease status.

Poisson regression was used to obtain the adjusted frequencies of dietary practices between individuals with or without the diagnosis of DM and/or SAH, throughout the “margin” command in Stata program. All sociodemographic variables with p-value <0.20 in the bivariate analysis were included in the adjusted analyses as potential confounders. The value of statistical significance was set at 5%.

The EpiFloripa Adults 2009 project was approved by the Ethics Committee on Human Research of the Federal University of Santa Catarina (UFSC), under protocol number 351/08. The subjects were informed about the objectives of the study and were requested to sign an Informed Consent Form.

## Results

A total of 1720 adults (85.3% of the estimated sample) were effectively enrolled in the study. Most of the sample was female (55.8%), with white skin colour (84.2%) and had a mean of 38 years of age (SD = 11.6; 31.4% aged from 29 to 39 years). No differences were found between genders for socioeconomic variables (Table 1).

**Table 1:** **Sociodemographic and dietary practices variable description of 20–59 year-old adults from the EpiFloripa survey, stratified by gender (n = 1720)**

	**Male (n = 761)**		**Female (n = 959)**	
**Variable**	**n**	**% (CI95%)**	**n**	**% (CI95%)**
**Age in years**				
20 to 29	260	34.2 (30.7;37.5)	280	29.2 (26.3;32.1)
30 to 39	172	22.6 (19.6;25.6)	220	22.9 (20.3;25.6)
40 to 49	181	23.8 (20.8;26.8)	257	26.7 (24.0;29.6)
50 to 59	148	19.4 (16.6;22.3)	202	21.1 (18.5;23.6)
**Self-reported skin colour** ^**a**^				
White	642	84.5 (81.9;87.1)	802	84.0 (81.6;86.3)
Dark	74	9.7(7.6;11.8)	73	7.6 (6.0;9.3)
Black	34	4.5 (3.0;5.9)	53	5.5 (4.1;7.0)
Other	10	1.3 (0.5;2.1)	27	2.8 (1.7;3.9)
**Per capita family income (tertiles)** ^**a**^				
First tertile (Rich)	258	34.6 (31.2;38.6)	301	32.0 (29.0;35.0)
Second tertile (Intermediate)	258	34.6 (31.2;38.6)	304	32.3 (29.3;35.3)
Third tertile (Poor)	229	30.7 (27.4;34.1)	335	35.6 (32.6;38.7)
**Education level in years** ^**a**^				
12 or more	318	41.9 (38.4;45.5)	419	43.7 (40.6;46.9)
9 to 11	263	34.7 (31.3;38.1)	305	31.8 (28.9;34.8)
0 to 8	177	23.3 (20.3;26.4)	234	24.4 (21.7;27.2)
**Regular fruit consumption** ^**b**^				
Yes	245	32.2 (28.9;35.6)	487	50.1 (47.7;54.0)
No	515	67.8 (64.4;71.1)	471	49.1 (46.0;52.3)
**Regular vegetable consumption** ^**a,b**^				
Yes	547	75.4 (72.4;78.5)	802	83.7 (81.4;86.1)
No	187	24.6 (21.5;27.6)	156	16.3 (13.9;18.6)
**Fruit consumption ≥ 3 times/day** ^**a,b**^				
Yes	51	6.7 (4.9;8.5)	139	14.5 (12.3;16.7)
No	709	93.3 (91.5;95.1)	819	8.5 (83.2;87.7)
**Vegetable consumption ≥ 3 times/day** ^**a,b**^				
Yes	462	60.8 (57.3;64.3)	686	71.7 (68.8;74.5)
No	298	39.2 (35.7;42.7)	271	28.3 (25.5;31.2)
**Regular fat of meat consumption** ^**a,c,d**^				
No	484	63.7 (60.2;67.1)	758	79.1 (76.5;81.7)
Yes	276	36.3 (32.9;39.7)	200	20.9 (18.2;23.5)
**Regular fried food consumption** ^**a,c**^				
No	300	39.4 (35.9;42.9)	539	56.4 (53.2;59.5)
Yes	461	60.6 (57.1;64.1)	417	43.6 (40.5;46.8)
**Regular sugar-sweetened soda consumption** ^**a,c**^				
No	282	37.1 (33.6;40.5)	442	46.2 (43.0;49.4)
Yes	479	62.9 (59.5;66.4)	515	53.8 (50.6;57.0)
**Self-reported DM and/or SAH diagnosis** ^**a**^				
No	632	83.0 (80.4;85.7)	801	83.7 (81.3;86.0)
Yes	129	17.0 (14.3;19.6)	156	16.3 (14.0;18.6)

DM – diabetes mellitus; SAH – systemic arterial hypertension.

^a^Variables with missing data at most 0.3%, except for per capita family income (2%).

^b^≥ 6 times/week consumption.

^c^≥ 2 times/week consumption.

^d^Includes red meat fat and/or chicken skin.

With regard to the consumption of unhealthy food, in the overall sample more than 50.0% reported a regular consumption of fried foods (51.0%) and soda (57.9%). For meat fat, consumption was less frequent (27.7%). About the consumption of healthy foods, only 42.6% of the sample consumed fruit in a regular basis, while fruit consumption ≥ 3 times/day was found in 11.1%. In the case of vegetables, 80.0% reported consuming ≥6 times/week and 66.9% said they ate ≥2 servings/day (data not shown). Unhealthy food consumption was more common in men, while healthy food consumption was more frequent in women (p < 0.001 for all analyses) (Table 1). In turn, the prevalence of DM was 3.7% and SAH 14.8%, whereas the diagnosis of DM and/or SAH was confirmed by 16.6% of participants (no gender difference, p = 0.719).

**Figure 1:**
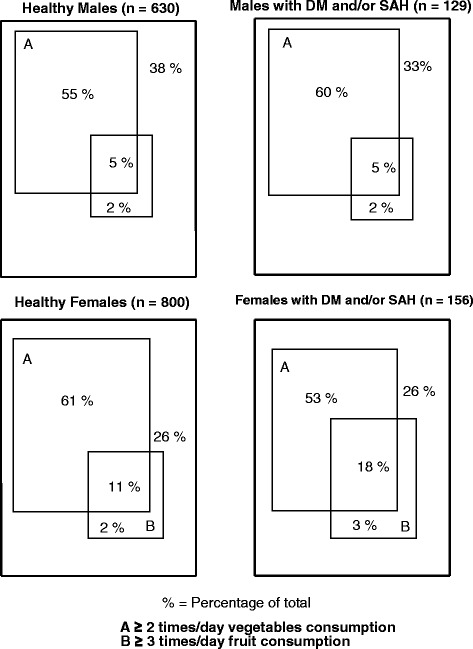
Venn diagram of dietary practices adoption related to healthy food consumption. Stratified by the presence or not of diabetes mellitus and/or systemic arterial hypertension and by gender. Florianópolis, Brazil, 2009.

In Figure 1, the Venn diagram shows the frequency of dietary practices in relation to consumption of healthy food (consumption of ≥ 2 times/day of vegetables and ≥ 3 times/day of fruit), depending on the health status of individuals (with or without DM and/or SAH), according to gender. Only 5.0% of healthy men and 11.0% of healthy women consumed both, fruit and vegetables; whereas among those with DM and/or SAH the respective frequencies were 5.0% and 18.0%. In turn, 38.0% of healthy men and 26.0% of healthy women reported not consuming adequate daily frequency of either food; while 33.0% of male and 26.0% of female diagnosed with DM and/or SAH showed the same pattern (P = 0.62 for men and P = 0.08 for women).

**Figure 2:**
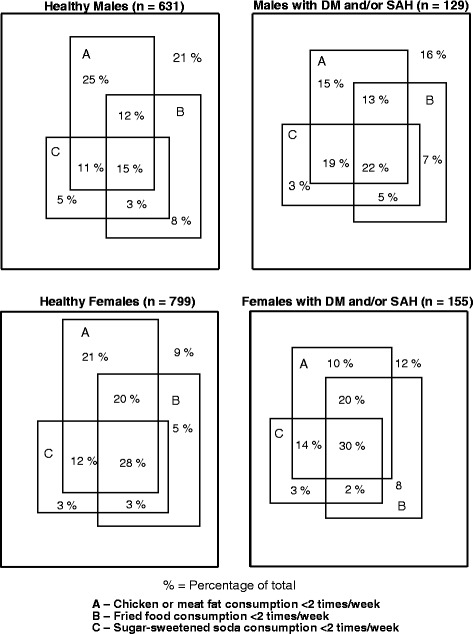
Venn diagram of dietary practices related to unhealthy food consumption. Stratified by the presence or not of diabetes mellitus and/or systemic arterial hypertension, and by gender. Florianópolis, Brazil, 2009.

Regarding unhealthy food dietary practices, the Venn graph (Figure 2) shows that 15.0% of healthy men and 28.0% of healthy women, as well as 22.0% and 30.0% of the diagnosed participants had proper dietary practices for these types of food, respectively. On the other hand, 21.0% of healthy men and 9% of healthy women reported consuming ≥ 2 times/week each unhealthy food (meat fat, fried foods and/or soft drinks), while the diagnosed participants reported 16 and 12%, respectively (P = 0.12 for men and P = 0.25).

**Figure 3:**
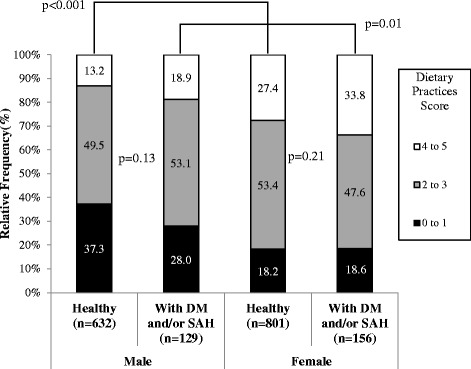
Dietary practices score of 20-59 year-old males and females living in Florianópolis, Brazil, 2009. Stratified by the presence or not of diabetes mellitus and/or systemic arterial hypertension, and by gender.

Figure 3 shows the scale of healthy dietary practices of respondents (consumption ≥ 3 times/day of fruit, ≥ 2 times/day of vegetables and consumption of at most 1 time/week meat fat, fried foods and/or soft drinks). The frequency of 4–5 healthy dietary practices was greater in women compared to men, regardless of whether or not they had DM and/or SAH (14.0% higher in healthy women compared to healthy men, and almost 15.0% higher in women with DM and/or SAH when compared to diagnosed men). Still, the frequency of 4–5 healthy dietary practices was not greater than 35%, even in females. In men, almost 87.0% of healthy individuals and 81.1% of diagnosed individuals showed a 0–3 healthy dietary practices rate, while women showed 71.6% and 66.2% (15.4% and 14.9% greater for men, respectively). Although gender differences were significant (p < 0.05), no differences were found for the number of dietary practices between healthy and diagnosed men and women (p > 0.10 in both cases).

Table 2 shows the crude and adjusted association between the presence or not of DM and/or SAH (exposure) and healthy/unhealthy dietary practices variables (outcome). In men, only the regular consumption of fried foods and soft drinks were lower in individuals with DM and/or SAH (17.0% and 21.0% lower, respectively) compared with healthy subjects. However, the association was completely confounded by sociodemographic variables. Among woman, in crude analysis, proper fruit consumption was 63.0% greater among those with DM and/or SAH than among healthy subjects, but this association disappeared after adjustment. And contrary to expected, consumption of meat fat was 57.0% greater among those diagnosed with DM and/or SAH compared with healthy individuals. However, this association became nonsignificant after adjustment for confounders. None of the other variables of consumption was associated with disease condition for both genders, neither in crude or adjusted analysis.

**Table 2:** **Crude and adjusted analysis of food consumption among people with and without chronic diseases (diabetes mellitus and/or hypertension) in adults aged 20-59 years included in the EpiFloripa study**

	**Analysis type**	**Male**			**Female**		
		**Healthy (n=632)**	**With DM and/or SAH (n=129)**	**p value**	**Healthy (n=801)**	**With DM and/or SAH (n=156)**	**p value**
		**% (CI95%)**	**% (CI95%)**		**% (CI95%)**	**% (CI95%)**	
**Regular fruit consumption** ^**a**^	**Crude**	30.4 (26.1-35.2)	39.8 (31.3-48.9)	0.06	48.2 (44.3-52.3)	58.5 (51.4-65.2)	0.007
	Adjusted*	32.0 (27.9-36.1)	32.2 (24.7-39.7)	0.96	50.4 (46.7-54.1)	48.3 (42.4-54.1)	0.51
**Regular vegetable consumption** ^**a**^	**Crude**	82.4 (78.2-86.0)	81.0 (73.0-87.0)	0.71	72.0 (68.0-75.7)	73.9 (65.8-80.7)	0.64
	Adjusted*	82.0 (78.5-85.5)	82.9 (76.3-89.4)	0.82	71.3 (67.9-74.7)	76.5 (69.1-84.0)	0.23
**Daily fruit consumption (≥3 times/day)** ^**a**^	**Crude**	6.1 (4.2-8.7)	6.9 (3.8-12.3)	0.70	12.6 (10.2-15.6)	20.6 (15.2-27.2)	0.007
	Adjusted*	6.4 (4.2-8.7)	6.2 (2.2-1.0)	0.92	13.2 (10.7-15.7)	16.4 (11.3-21.5)	0.25
**Daily vegetable consumption (≥2 times/day)** ^**a**^	**Crude**	59.2 (54.4-63.9)	63.7 (55.9-70.9)	0.32	71.4 (67.1-75.3)	72.8 (66.1-78.6)	0.70
	Adjusted*	61.8 (57.8-65.7)	56.2 (49.5-62.9)	0.14	71.9 (68.4-75.3)	67.9 (61.3-74.5)	0.29
**Regular fat of meats consumption** ^**b,c**^	**Crude**	25.4 (20.9-30.5)	25.9 (19.0-34.2)	0.90	10.8 (8.7-13.3)	17.0 (11.4-22.7)	0.02
	Adjusted*	26.1 (21.7-30.6)	23.8 (17.3-30.4)	0.50	10.8 (8.7-13.0)	15.4 (9.8-21.0)	0.09
**Regular fried food consumption** ^**b**^	**Crude**	62.3 (58.6-65.9)	51.4 (43.1-59.6)	0,016	44.3 (40.8-47.9)	40.3 (32.9-48.3)	0.33
	Adjusted*	60.2 (56.3-64.1)	57.4 (48.2-66.8)	0.60	42.6 (39.3-46.0)	47.4 (38.4-56.5)	0.31
**Regular sugar-sweetened Soda consumption** ^**b**^	**Crude**	64.8 (59.0-70.2)	51.5 (42.9-60.0)	0,006	54.0 (49.2-56.8)	49.8 (41.1-58.5)	0.34
	Adjusted*	63.2 (58.3-68.1)	58.2 (49.6-66.7)	0.34	53.0 (49.2-56.8)	55.5 (47.1-63.9)	0.61

Stratified by gender.

^a^≥6 times/week consumption.

^b^≥ 2 times/week consumption.

^c^Includes red meat fat and/or chicken skin.

*Adjusted for skin colour, age, per capita family income and education level.

## Discussion

This paper assessed in a population-based sample whether the different dietary practices – which are essential in the treatment and prevention of DM and SAH consequences [2,12,26] – were more frequent among people diagnosed with such NCDs, in comparison with the rest of the population. Based on the results, three main conclusions can be highlighted. First, whether in relation to healthy or unhealthy food consumption, healthier dietary practices were more frequent in women than men. Second, no differences were found for dietary practices when comparing those diagnosed with DM and/or SAH with the rest of the sample, even among women. Third, the few results that pointed to better dietary practices of individuals with DM and/or SAH compared to healthy individuals were completely confounded by sociodemographic variables, especially after adjustment for education level. Therefore, our results suggest that sociodemograhic variables, in particular education level, are more important for dietary practices than the presence of DM and/or SAH. This is particularly relevant for public health promotion, as previous studies have also shown that gender, family income and education level are deeply related to food habits [23,27-29]. The lower the income and education level, the lower the daily intake of fruits and vegetables, and the higher the consumption of fatty foods. Additionally, socioeconomic position and gender are also related to health services access, and in consequence, to the diagnosis and treatment of chronic diseases [24].

In this study, women consumed fruit and vegetables more frequently, and had a lower intake of meat fat, fried foods and soft drinks compared to men. This result is consistent with evidence from previous studies showing that women not only take more care on their dietary practices, but also have a higher utilization of health care services when compared to men [22-24]. Even the increased demand for health services may result in greater diagnosis for women (detection bias); prevalence rates of both NCDs combined were similar in both genders, which reduces the probability that this bias has affected the results. Nevertheless, the matters listed above justify the need for stratification of analyses by gender.

In turn, the use of individual’s report on the diagnosis of DM and/or SAH may underestimate the actual prevalence of these diseases, considering that this type of self-reported information has approximately 70% sensitivity and 90% specificity [30]. In our study, prevalence rates for both diseases (3.7% for DM and 14.8% for hypertension) were lower than available estimates to this age group (6-7% and 30%, respectively) [31]. Nevertheless, it is unlikely that these underestimates affected the association of diseases with the different dietary practices, since changes in food intake would be expected only among individuals that were aware of their diagnosis.

Several international [2,4,13] and Brazilian entities [6] recommend a diet rich in fruits and vegetables, low in saturated and total fat, and reduced consumption of sugary drinks, as primary and secondary prevention measures in fighting against obesity and NCDs. Healthy dietary practices may be responsible for a reduction of up to 5–6 mm Hg in systolic and up to 3 mmHg diastolic [13] blood pressure, and may help to reduce the conversion rates of insulin resistance to type 2 diabetes by up to 43% in a period of twenty years [2,14]. Despite this evidence, and that the existing recommendations should be followed by the entire population, three of the healthy dietary practices examined have much lower frequency than expected, even among people diagnosed with DM and/or SAH. In both genders and regardless of disease condition, the frequency of daily fruit intake did not exceed 20%. In turn, regular consumption of fried foods and sodas ranged from 40-60% (higher in men than in women). This scenario has also been evidenced in high-income countries. The NHANES study, conducted in the United States with 7,811 40–74 year-old individuals, assessed from 2001–2006, showed that only 26% of participants consumed five or more servings of fruits and vegetables, and individuals with history of DM, SAH and/or cardiovascular disease showed no differences in dietary practices compared to healthy subjects [32].

These percentages above are worrisome, considering the changes in the profile of food habits for the Brazilian population. When assessing the changes in the consumption of sodas in state capitals, data from the Survey of Family Budgets (*Pesquisa de Orçamentos Familiares*) [33] shows that, among the surveys of 2002–2003 and 2008–2009 there was a 39% increase in the purchase of these products in the urban area and 92% in rural areas. In the same period, despite an increase of 17.9% on purchases of fruits, vegetables decreased by approximately two kilograms per capita. In addition, the lack of healthy dietary practices affects not only the individuals, but the entire Brazilian health system. Brazil estimates a total spend of US$ 398.9 million every year for the treatment of hypertension in the public health system, which represents 1.43% of the total expenditures of the Unified Health System (SUS) [10]. These costs are even greater when considering the associated complications, such as cardiovascular disease. The latter have accounted for almost 1.2 million hospitalizations in Brazil in 2005, which cost US$ 546.6 million to the Brazilian government [10].

The low frequency of healthy dietary practices mentioned in this study was also evident when consumption was analysed in a healthy dietary practices score. Only 13.2% of men and 33.8% of women had 4–5 points, with no difference between healthy and DM and/or SAH diagnosed groups. A cross-sectional population-based study conducted in the United States assessed the adequacy of Dietary Approaches to Stop Hypertension (DASH) in 5,867 adults, considering a score of up to nine points [15]. That study found no difference in dietary practices among people without DM, with DM and SAH or only with DM.

Several interventions to encourage the consumption of healthy foods, as well as other health care actions have been carried out, but showed poor results, often improving the knowledge, but not the healthy behaviours [34]. On the other hand, the literature also indicates deficiency in the quality of information passed on by health professionals to DM and/or SAH patients in relation to lifestyle changes. While treatment guidelines for these diseases do recommend changes in lifestyle as the first therapeutic measure for secondary prevention, health professionals use the prescription of antihypertensive medications and/or hypoglycemic agents as initial treatment for these patients [35,36].

According to Santos & Victora [37], the reasons for the low effectiveness in primary health care include a long causal chain that starts in carrying out interventions and ends on their consequences, the functioning of the health system (access, availability of supplementary exams and drugs), besides environmental, cultural, demographic and epidemiological matters. Meanwhile, developing healthy dietary practices is a difficult task, because unhealthy habits are associated with feelings of pleasure, and are widely disseminated in the media, and they show harmful effects in a slow and quiet manner [1,38].

Some national actions and policies have been developed with the intent of modifying eating habits and reducing the prevalence of NCDs in the Brazilian population, such as the “Strategic Action Plan for the Fight Against NCDs in Brazil , 2011-2022” released by the Ministry of health in 2011 [26] and the “National Food Policy at School”, which encourage healthy eating habits from the earliest years of life [39]. Such initiatives are critical for the success of the primary and secondary prevention of these diseases and other NCDs, considering the need for early guidance on these issues. It is also crucial to educate different sectors of society about the importance of dietary practices, expanding the reach of prevention and reducing the development of NCDs and their complications. Further research is needed to assess the effectiveness of these policies on health, not only in relation to the prevalence of these diseases, but also in changing the lifestyle of the population.

A possible limitation of this study was the use of a semiquantitative questionnaire to assess dietary practices, even though it was based on an instrument employed in periodic national surveys regarding the health status of the population [19]. Another limitation related to dietary practices is the lack of analysis of other foods considered harmful for the diseases investigated, such as processed and ultra-processed foods (high in sodium, saturated fat and sugars). Finally, the cross-sectional design used in the study doesn’t allow drawing conclusions about the cause-effect between the NCDs diagnosis and changes in dietary practices. However, this study based on a representative sample of the population of Florianópolis is important, as it allows to reproduce the population structure of the municipality, ensuring the external validity of the research. Moreover, several procedures were adopted in this study to improve data quality, both in data collection and analysis.

## Conclusions

To conclude, our results showed low frequency of healthy dietary practices for both healthy and DM and/or SAH diagnosed individuals. Even though the lack of healthy dietary practices in the general population is an aggravating factor for the increased prevalence of obesity and NCDs in a middle-income country such as Brazil, it is more worrisome that DM and/or SAH are not associated with healthy dietary practices, since the morbidity and mortality associated with these diseases have a major impact on the individual, their families and society. Thus, the challenge for public health is not only to investigate the reasons for the lack of meticulous care, but also to create health policies and comprehensive educational programs to encourage a change on the eating behaviour of the population.

## Abbreviations

DM: Diabetes mellitus
NCD’s: Non-communicable diseases
NHANES: National Health and Nutrition Examination Survey
SAH: Systemic arterial hypertension
SUS: Unified Health System
WHO: World Health Organization
